# Genetic association of *rs1344706* in *ZNF804A* with bipolar disorder and schizophrenia susceptibility in Chinese populations

**DOI:** 10.1038/srep41140

**Published:** 2017-01-25

**Authors:** Shuquan Rao, Yao Yao, Joanne Ryan, Chunhui Jin, Yong Xu, Xinhe Huang, Jianxiu Guo, Yueqiang Wen, Canquan Mao, David Meyre, Fuquan Zhang

**Affiliations:** 1 School of Life Science and Engineering, Southwest Jiaotong University, Chengdu, 610031, China; 2 Department of Fundamental Medicine, Chengdu University of Traditional Chinese Medicine, Chengdu, 611137, China; 3 Disease Epigenetics Group, Murdoch Children’s Research Institute, & Department of Paediatrics, University of Melbourne, 3052 Parkville, Australia; 4 Inserm, U1061, Univ Montpellier, Montpellier, France; 5 Wuxi Mental Health Center, Nanjing Medical University, Wuxi, China; 6 Department of Psychiatry, First Clinical Medical College/First Hospital of Shanxi Medical University, Taiyuan, 030000, China; 7 Department of Clinical Epidemiology and Biostatistics, McMaster University, Hamilton, ON L8N 3Z5, Canada

## Abstract

*Rs1344706* in the the zinc finger protein 804A (*ZNF804A*) gene has been identified to be associated with schizophrenia and bipolar disorder (BD) in Europeans. However, whether *rs1344706* is associated with schizophrenia in Chinese populations remains inconclusive; furthermore, the association between *rs1344706* and BD in Chinese populations has been rarely explored. To explore the association between *rs1344706* and schizophrenia/BD in Chinese populations, we genotyped *rs1344706* among 1128 Chinese subjects (537 patients with BD and 591 controls) and found that r*s1344706* showed marginal allelic association with BD (*P* = 0.028) with T-allele being more prevalent in cases than that in controls (OR = 1.19, 95% CI 1.03–1.37). Meta-analysis of *rs1344706* by pooling all available data showed that *rs1344706* was significantly associated with BD (*P* = 0.001). Besides, positive association of *rs1344706* with schizophrenia was observed in Northern Chinese (*P* = 0.005). Furthermore, *ZNF804A* is highly expressed in human and mouse brains, especially in prenatal stage.

Schizophrenia and bipolar disorder (BD) are two severe neuropsychiatric disorders, with each affecting approximately 1% populations worldwide^1^. Evidence from family, twin and adoption studies highlights the pivotal role of genetic components in the two disorders, with a heritability estimated at 80–85% for schizophrenia^2^ and 85–89% for BD^3^.

Though schizophrenia and BD have been defined as separate mental disorders over the past 100 years, both disorders share some clinical symptoms with each other, *i.e*., psychosis in BD and affective disruption in schizophrenia as observed in clinical practice^4^, suggesting shared pathophysiological mechanisms between schizophrenia and BD. Corresponding to this observation, increasing number of studies have revealed an overlap of risk variants between schizophrenia and BD^5^.

Till now, several dozens of risk single nucleotide polymorphisms (SNP) and genomic regions which showed significant association with both schizophrenia and BD have been identified through candidate gene association study (*rs1006737* in *CACNA1C* and rs10994336 in ANK3)^6^,^7^, and genome-wide association studies (GWAS) (*rs1109803* in *NDST3*)^8^.

Another promising common risk variant for schizophrenia and BD is *rs1344706* in the zinc finger protein 804A (*ZNF804A*) gene, which was first identified by a GWAS in samples of Caucasian ancestry^9^. Follow-up studies conducted in different European populations have replicated this finding, suggestive that *rs1344706* is probably an authentic risk SNP for schizophrenia and BD in Europeans^10^,^11^. In Chinese populations, several groups have reported the association between *rs1344706* and schizophrenia, subjects from which were all from northern China (Shanxi, Xinxiang and Shandong)^12^,^13^,^14^. However, more studies with subjects recruited from central and Southern China (*i.e*., Jiangsu, Yunnan, Sichuan and Guangxi province) failed to replicate this association^15^,^16^,^17^,^18^. Whether *rs1344706* in the *ZNF804A* gene is associated with schizophrenia therefore remains inconclusive. To address this issue, meta-analyses were performed in Chinese populations and Asian populations by multiple groups; however, the results from different groups remain still elusive, due to limited sample size and different inclusion criteria of eligible studies^19^,^20^. Moreover, in contrast to the extensive exploration of the association between *rs1344706* and schizophrenia in Chinese populations, whether *rs1344706* confers risk to BD in Chinese remains largely unknown, although nominal association between *rs1344706* and BD has been observed recently by Zhang *et al*. in Shanghai (*P* = 0.034, OR = 1.19), with a relatively small sample (746 patients of BD and 762 controls)^21^.

In this study, we first explored the association between *rs1344706* and BD in our Chinese sample consisting of 537 cases and 591 controls, and then conducted a meta-analysis to test the association of *rs1344706* with either schizophrenia (11573 cases and 15321 controls, as well as 101 schizophrenia trios) or BD (1274 cases and 1343 controls) in Chinese populations with the largest sample size available to date. Moreover, temporal and spatial expression patterns of *ZNF804A* in different human brain regions were investigated using available online database.

## Results

### *Rs 1344706* T-allele was marginally associated with BD in our samples

The genotypic distribution of *rs1344706* confirmed to HWE in both control (χ^2^ = 0.29, *P* = 0.59) and case (χ^2^ = 2.41, *P* = 0.12) groups. As shown in Table 1, *rs1344706* showed marginal allelic association with BD in our samples (χ^2^ = 4.85, *P* = 0.028), with T-allele more prevalent in patients of BD than that in healthy subjects (OR = 1.21, 95% CI 1.02–1.43), which survived after adjusting for age and gender (*P* = 0.047). Moreover, there was no significant difference in genotype frequencies of *rs1344706* between the case and control group (χ^2^ = 5.62, *P* = 0.060). We further analyzed the association of *rs1344706* with BD by gender, which revealed significant allelic association between *rs1344706* T-allele and BD in males (χ^2^ = 4.48, *P* = 0.034, OR = 1.33, 95% CI 1.02–1.74) but not in females (*P* > 0.05). The association of *rs1344706* with disease characteristics, *i.e*., age of onset, depressive or manic episodes, were summarized in Table S1.

The present sample size (537 BD cases and 591 controls) revealed approximately 32.6% power of detecting a significant association for *rs1344706* with the following assumptions: *P* = 0.05, OR = 1.20 corresponding to a “weak to moderate” effect, and a given MAF of 0.453 according to the 1000 Genomes project (www.1000genomes.org/) (the same bellow).

### Meta-analysis of the association of rs1344706 with BD and schizophrenia

To obtain a more comprehensive interpretation of the association of *rs1344706* with psychiatric disorders, mainly BD and schizophrenia, we performed a meta-analysis by pooling our data and those from published studies conducted in Chinese populations. Following our literature search strategy, a total of 11 studies involving 15 independent samples which encompass 11573 cases and 15321 controls, as well as 101 schizophrenia trios, were identified for schizophrenia^9^,^12^,^13^,^14^,^15^,^16^,^17^,^18^,^22^,^23^,^24^, and 2 studies (including the present study) consisting of 1274 patients of BD and 1343 healthy controls was identified for BD^21^. Detailed information concerning the sample size and association results for each study was listed in Table 2. As shown in Fig. S1, there was no evidence implying publication bias checked by the Egger regression test (*P* = 0.596) and the Begg–Mazumdar test (*P* = 0.235).

**Figure 1:**
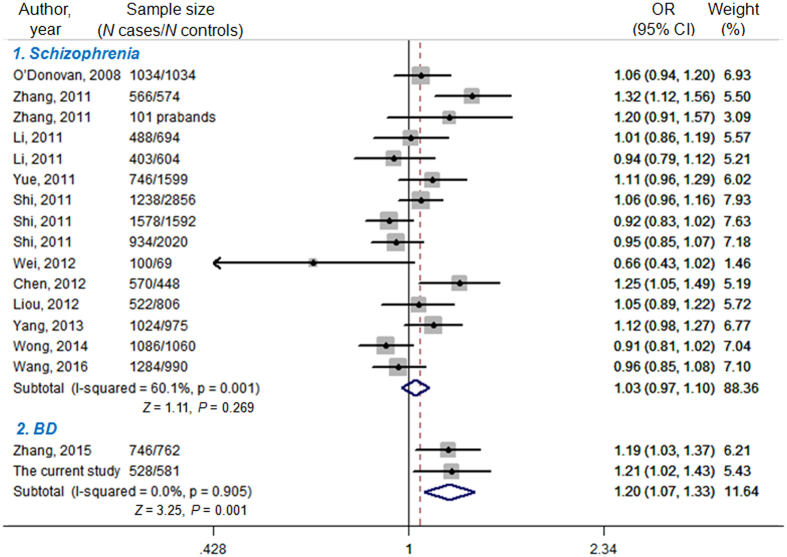
Meta-analysis for *rs1344706* T-allele of the *ZNF804A* gene in schizophrenia and BD. The sources of the published data were listed in Table 2. The random-effect model was applied to pool the data for association *rs1344706* and schizophrenia, while the fixed-effect model for BD.

Before the pooling procedure, statistical power was assessed given the *P* of 0.05, OR of 1.20 and MAF of 0.453. For schizophrenia, the pooled sample size revealed 100% power to detect a significant association of *rs1344706*; for BD, the pooled sample size could provide 64.2% power to detect a significant association. Meta-analysis showed that there was significant association between *rs1344706* T-allele and BD (*P* = 0.001, OR = 1.20, 95% CI 1.07–1.33) by the fixed-effect model, since no significant evidence of between-study heterogeneity was observed (I^2^ = 0.0%, *P* = 0.905). However, we observed no significant difference in T-allele distribution of *rs1344706* between schizophrenia and controls (*P* = 0.269, OR = 1.03, 95% CI 0.97–1.10) using the random-effect model due to significant evidence of between-study heterogeneity (I^2^ = 60.1%, *P* = 0.001) (Fig. 1).

After careful examination of the regional distributions of recruited populations, we found that most of them resided in Northern China, followed by Southern and Central China. Moreover, the MAF of *rs1344706* in controls was slightly different among Northern (0.489)^13^, Central (0.41)^9^ and Southern (0.491)^15^ Chinese populations, which might cause between-study heterogeneity. Therefore, we conducted meta-analysis using studies from northern, central, and southern China respectively. As shown in Fig. S2, significant association between *rs1344706* T-allele and schizophrenia (*P* = 0.005, OR = 1.09, 95% CI 1.03–1.15) was observed in Northern Chinese populations by the random-effect model, but not in Central (*P* = 0.357) or Southern (*P* = 0.132) Chinese populations by the fixed-effect model.

### *Rs1344706* showed dramatic allelic difference in global populations

Recent studies suggest that genetic variants conferring risk of psychiatric disorders show significant frequency differences among worldwide populations, such as *rs2709373* in *CREB1*^25^ and *rs6001946* in *MKL1*^26^. Therefore, we detailed the global allele frequency distributions of *rs1344706* in 53 world populations using the HGDP database^27^ and 1000 Genomes project^28^. Intriguingly, the derived allele (C-allele) of *rs1344706* showed dramatic frequency differences among world populations, with the highest frequencies in most Asian populations, followed then by Middle East and Europe. At the extremes, we found that *rs1344706* is nearly fixed for ancestral allele (A-allele) in most African populations (Fig. S3).

### Spatiotemporal expression patterns of *ZNF804A* in different human brain regions

**Figure 2:**
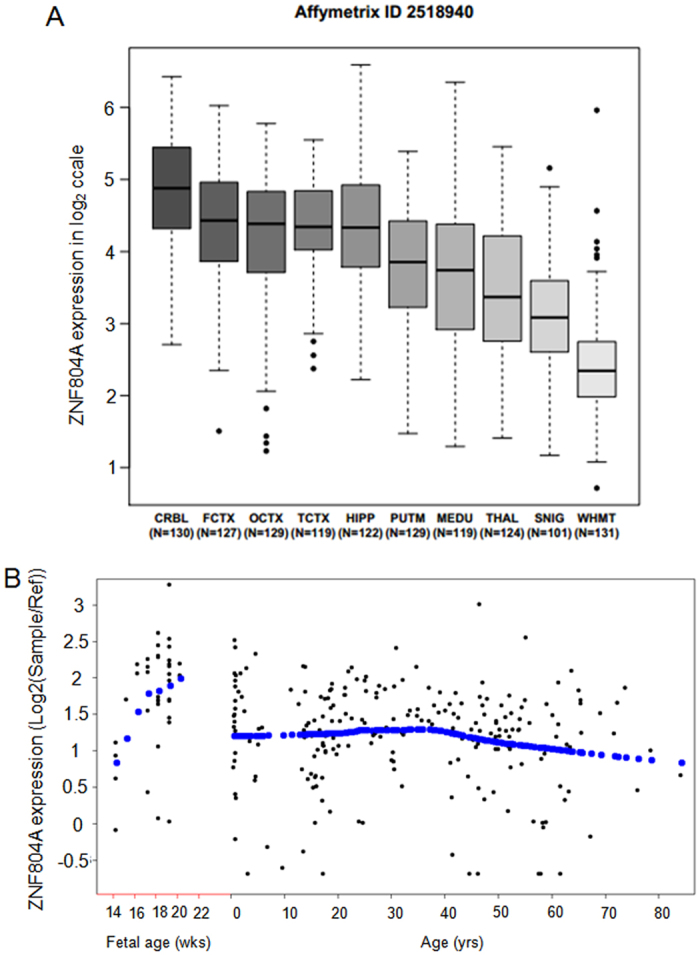
Spatiotemporal expression pattern of the *ZNF804A* gene in human brain regions. (**A**) *ZNF804A* is expressed in various brain regions, with the highest transcript level in cerebellar cortex (from the BRAINEAC). CRBL, cerebellar cortex; FCTX, frontal cortex; OCTX, occipital cortex; TCTX, temporal cortex; HIPP, hippocampus; PUTM, putamen (at the level of the anterior commissure); THAL, thalamus (at the level of the lateral geniculate nucleus); MEDU, inferior olivary nucleus (sub-dissected from the medulla); SNIG, substantia nigra; WHMT: intralobular white matter. (**B**) Temporal expression profiling of *ZNF804A* in the human PDFC of normal subjects across lifespan (from the BrainCloud).

To further test whether *ZNF804A* contributes risk to BD, we explored the expression profiling of *ZNF804A* in diverse human brain tissues using the BRAINEAC data^29^. We found that *ZNF804A* is expressed in various brain regions, with the highest transcript level in cerebellar cortex, followed by frontal cortex, occipital cortex and temporal cortex (Fig. 2A). Using the expression data from BioGPS^30^, we confirmed that *ZNF804A* is mainly expressed in brain tissues, with limited or no expression in peripheral tissues (Fig. S4). Besides, we examined the temporal expression pattern of *ZNF804A* in developing and adult DPFC of normal subjects using the BrainCloud^31^. The expression of *ZNF804A* is relatively high in prenatal period, especially the late developmental stage (>20 weeks) (Fig. 2B). After birth, *ZNF804A* expression level is decreased, suggesting that ZNF804A plays a crucial role in brain development and dysregulation of ZNF804A may then confer risk to BD.

## Discussion

Recently, *rs1344706* in the *ZNF804A* gene was implicated as one of the most compelling genetic loci that contribute to the susceptibility of both schizophrenia and BD in European populations, supported by the GWAS and follow-up replications. In the present study, we detected significant association between *rs1344706* T-allele and BD in Chinese samples both in our case-control cohorts and subsequent meta-analysis. It should be noted that, as the sample size increased, this association between *rs1344706* and BD was strengthened as indicated by sharply reduced *P*-value (seen in the meta-analysis), suggesting that *rs1344706* may serve as an authentic risk SNP for BD in Chinese populations. Moreover, stratified analysis by gender showed that *rs1344706* was associated with male BD (*P* = 0.034) but not with female BD (*P* = 0.218) in our samples, which was consistent with previous studies^32^. However, this association observed for *rs1344706* with BD should be treated scrupulously, since the sample size used in the present study was underpowered which might inflate potential false positive associations and even drew an opposite conclusion. We noticed that our in-house samples (537 BD cases and 591 controls) revealed approximately 32.6% power of detecting a significant association for *rs1344706*, and even by combining all available data, the sample size (1274 patients with BD and 1343 controls) could only provide 64.2% power to detect a significant association.

Intriguingly, the allelic frequency of *rs1344706* showed dramatic difference among worldwide populations, suggestive its implication in psychiatric disorders. From the aspect of evolution, the prevalence of specific disorders should decrease with time, however, this is not the case for psychiatric disorders which maintain a stable or more prevalence across all human cultures. One potential explanation for this paradox suggests positive selection as the driving force in maintaining risk loci of psychiatric disorders in the gene pool. In line with the hypothesis, genetic variants conferring risk of psychiatric disorders might show significant frequency differences among worldwide populations, and several risk loci of psychiatric disorders were thus identified, including *rs2709373* in *CREB1*^25^ and *rs6001946* in *MKL1*^26^.

In addition, our meta-analyses indicated significant association of *rs1344706* with schizophrenia in Northern Chinese populations (*P* = 0.005, OR = 1.09, 95% CI 1.03–1.15), *i.e*., Shanxi, Xinxiang and Shandong^12^,^13^,^14^, which was inconsistent with most studies conducted in either Central or Southern Chinese populations, including Jiangsu, Yunnan, Sichuan and Guangxi province^15^,^16^,^17^,^18^. This inconsistence may originate from the following several reasons. First, divergent genetic backgrounds in different regions may result in differentiation in allele frequencies and linkage disequilibrium (LD) patterns, which can then lead to inconsistent associations. As reported in previous studies, the MAF of *rs1344706* in controls was slightly different among Northern (0.489)^13^, Central (0.41)^9^ and Southern (0.491)^15^ Chinese populations. Second, differentiated environmental exposure, dietary and cultures might also contribute to inconsistent associations among Northern, Central and Southern Chinese, since incidence of schizophrenia can be greatly influenced by risk factors other than genetic variants. Given the relatively large sample size used, we thought that *rs1344706* was a risk variant for schizophrenia in Northern Chinese populations, but not in Central or Southern Chinese populations.

*ZNF804A*, consisting of four exons and three introns (*rs1344706* in intron 2), is located on chromosome 2q32.1 and potentially encodes a transcription factor. It has been shown that T-allele of *rs1344706* can weaken the binding affinity of unidentified nuclear protein with ZNF804A in neural cells, indicating that *rs1344706* may have a direct effect on *ZNF804A* expression and transcriptional regulation of its target genes^33^. Supporting it, Zhang *et al*. demonstrated that *rs1344706* T-allele was significantly associated with elevated expression level in both the occipital cortex and hippocampus via the BRAINEAC database^21^. Besides, we noticed that ZNF804A has peak expression level during fetal developmental stage, suggesting that *rs1344706* may confer susceptibility for BD by influencing early brain development. Finally, we found that ZNF804A was mainly expressed in cortical regions, followed by hypothalamus. In previous studies, abnormal cortical structures and functions have been extensively reported in BD patients. For example, BD patients exhibited significantly decreased thickness and increased activation in occipital cortex compared with healthy subjects, as demonstrated by MRI^34^.

However, there are several limitations to our interpretation of this study. First, the sample size in our initial case-control cohort was relatively small (528 patients of BD and 581 controls). Even when pooled with previously published data, the sample size (1274 cases and 1343 controls) was still modest. In order to exclude the possibility of false-positive, replication studies in more Chinese samples from different regions are required. Second, only one SNP, *rs1344706*, was analyzed in our samples, and SNPs located in other LD blocks and rare variants were not studied, which might miss some important genetic information.

In summary, we observed significant association of *rs1344706* in the *ZNF804A* gene with BD and schizophrenia, especially in Northern Chinese populations. However, the association between *rs1344706* and BD susceptibility should be interpreted with caution as a result of the limited sample size used in the present study and hereby produced false positive associations. Further investigations in larger samples are warranted to draw a solid conclusion.

## Methods

### Participants, DNA sampling and genotyping in our samples

In the present study, we recruited a total of 537 unrelated patients with BD type I or type II and 591 healthy subjects. All participants were of the Han Chinese origin and geographically came from the South of China. The patients who met DSM-IV criteria for BD type I or type II were recruited from outpatients that were admitted to the department of Psychiatry, the Affiliated Hospital of Southwest Medial University between June 2011 and January 2014.

All patients underwent the Structured Clinical Interview for DSM-IV-TR Axis I Disorders (SCID-I) -Patient Edition independently by two experienced psychiatrists. Meanwhile, the Hamilton Rating Scale for Depression-17 (HRSD-17) and Young Mania Rating Scale (YMRS) were used to assess depressive and manic features, respectively. Subjects with co-morbid diagnosis of other psychiatric disorders or severe organic disease were excluded from this study. The Extensive Clinical Interview contains items to assess demographics, ages at onset, episodes (depressive and manic) and mental status for the patients. Meanwhile, healthy controls were recruited from local communities. The detailed description of patients and controls were seen in Table S2.

Genomic DNA was extracted from peripheral blood leukocytes according to the standard phenol/chloroform procedure. *Rs1344706* was genotyped by a TaqMan method as described in our previous study^35^. To assess the genotyping error rate, approximately 10% samples were randomly selected and retested, and the genotype concordance was 100%. The genotype call rate for *rs1344706* was 98.3%.

Each subject provided written informed consent to attend this study

### Meta-analysis of the association of rs1344706 with BD and schizophrenia

Meta-analysis was performed in agreement with previously described methods^36^,^37^,^38^. Eligible studies included in the analysis were selected from PubMed, Embase, Medline, ISI Web of Knowledge, ScienceDirect and SCOPUS database by searching the following keywords: zinc finger protein 804A gene or *ZNF804A; rs1344706*, SNP or common variants; schizophrenia; bipolar disorder, BD or BPD in varying combinations. Eligible studies included in our meta-analysis must meet the following criteria: 1) manuscripts written in English; 2) study design being case-control or family-based association studies; 3) subjects being of Chinese ancestry; 4) commonly acceptable diagnosis criteria for patients, such as the Diagnostic and Statistical Manual of Mental Disorders (DSM); 5) available odds ratio (OR) with 95% confidence interval (95% CI), or sufficient data to calculate these statistics; 6) the genotypic frequencies of *rs1344706* in Hardy–Weinberg equilibrium (HWE) in healthy controls. Authors were contacted in case that the genotypic data was unavailable or there were other queries regarding their studies. Two independent authors extracted the following data from the main text: 1) author and publication year; 2) sample size, sample origin and genotyping method; 3) samples’ clinical status; 4) genotypic data of rs1344706 in both cases and controls; 5) *P*-value, OR and 95% CI.

For those samples used twice or more in different studies by the same group, only the studies with the largest sample size were included for analysis. Three samples^39^,^40^,^41^ were, therefore, excluded from the final meta-analysis as a result of partial overlap with other larger samples^13^,^17^. Besides, several genome-wide association studies (GWAS) of schizophrenia conducted with Chinese populations (Beijing, Bio-X in Shanghai and Taiwan) were also included in our meta-analysis^16^,^22^,^23^,^24^. We extracted the data of *rs1344706* from Huang *et al*. study^20^.

### Population genetic analysis of *rs1344706*

The allelic frequency distributions of *rs1344706* in global populations were derived from the Human Genome Diversity Project (HGDP) selection browser (http://hgdp.uchicago.edu/cgi-bin/gbrowse/HGDP/)^27^, which contains the alelle frequency data of SNPs in 53 worldwide populations, and the 1000 Genomes Project (www.1000genomes.org/)^28^.

### Spatiotemporal expression patterns of *ZNF804A* in different human brain regions

We detected the expression pattern of *ZNF804A* in diverse tissues and developmental stages of human brains using the following three data sets. 1) BRAINEAC (www.braineac.org/)^29^, a large exon-specific eQTL database covering ten brain regions from 134 post-mortem brains from individuals of European descent free from known neurological disorders. We used this database to explore spatial dynamics of *ZNF804A* expression in all covered brain regions. 2) BrainCloud^31^, which contains genome-wide genotyping data and whole transcriptome profiling data from postmortem dorsolateral prefrontal cortex (DPFC) of 269 healthy human subjects of different ages. This database aims to depict the temporal dynamics of gene expression across the lifespan, i.e., from 14 weeks at embryonic period through ageing. 3) BioGPS (http://biogps.org/)^30^, is a centralized gene portal, which contains information of gene expression data from 79 human and 61 mouse tissues. We explored the spatial expression pattern of *ZNF804A* in mouse. More detailed description of these data sets can be found in the original manuscripts.

### Statistical methods

We applied the χ^2^ goodness-of-fit test to estimate the Hardy-Weinberg equilibrium (HWE) for the genotypic distributions of *rs1344706* in our samples. Analysis of allelic and genotypic associations was performed by either the UNPHASED program (version 3.1.4) as used in our previous studies^35^. Regression analyses were applied to assess the association of rs1344706 allele distribution and disease characteristics. The odds ratio (OR) and 95% confidence interval (95% CI) were used to present the effect size of *rs1344706* T-allele in all analyses. We applied the Power and Sample Size Program software to perform power analysis, and the Stata12.0 statistical software package (http://www.stata.com/) to conduct publication bias analysis and meta-analysis. Degree of potential publication bias was checked using the Egger regression test for a funnel plot and the Begg–Mazumdar test. Cochran’s χ^2^-based Q-statistic was performed to assess the heterogeneity between individual OR estimates, which is considered significant at *P* < 0.10. When heterogeneity was present, the random-effects model was used to combine the OR; otherwise, the fixed-effect model was used. The significance of the pooled OR was determined by the Z test and *P* < 0.05 was considered statistically significant.

The study was approved by the Institutional Reviewing Board of Southwest Jiaotong University. All the procedures were in compliance with the Declaration of Helsinki and other relevant national and international rules.

## Additional Information

**How to cite this article:** Rao, S. *et al*. Genetic association of *rs1344706* in *ZNF804A* with bipolar disorder and schizophrenia susceptibility in Chinese populations. *Sci. Rep.* **7**, 41140; doi: 10.1038/srep41140 (2017).

**Publisher's note:** Springer Nature remains neutral with regard to jurisdictional claims in published maps and institutional affiliations.

## Supplementary Material

Supplementary Materials

## Figures and Tables

**Table 1:** Allelic and genotypic association of *rs1344706* with BD in our samples.

Group		Sample (N)	Allele frequency (%)		*P*-value	OR (95% CI)	Genotype frequency (%)			P-value
Total			T	G			TT	TG	GG	
	BD	528	581 (55.0)	475 (45.0)	**0**.**028**	1.21 (1.02–1.43)	151 (28.6)	279 (52.8)	98 (18.6)	0.060
	Control	581	585 (50.3)	577 (49.7)			144 (24.8)	297 (51.1)	140 (24.1)	
Male										
	BD	210	239 (56.9)	181 (43.1)	**0**.**034**	1.33 (1.02–1.74)	66 (31.4)	107 (51.0)	37 (17.6)	0.091
	Control	231	230 (49.8)	232 (50.2)			58 (25.1)	114 (49.4)	59 (25.5)	
Female										
	BD	318	344 (54.1)	292 (45.9)	0.218	1.15 (0.92–1.42)	87 (27.3)	170 (53.5)	61 (19.2)	0.412
	Control	350	355 (50.7)	345 (49.3)			86 (24.6)	183 (52.3)	81 (23.1)	

**Table 2:** Characteristics of included studies of *rs1344706* with schizophrenia in Chinese populations.

Author, year	Diagnostic method	Region	Case			control			*P*-value	OR	95(%) CI
			Sample (N)^1^	Male (%)	Age (mean, SD)	Sample (N)	Male (%)	Age (mean, SD)			
**Schizophrenia**											
**O’Donovan**^9^	DSM-IV	Shanghai	1034	55.1	38.8 (14.1)	1034	50.5	30.0 (8.7)	0.166	1.06	0.94–1.20
Zhang^12^	DSM-IV	Shanxi	566	52.1	34.0 (12.8)	574	57.3	29.1 (13.8)	8.30E-04	1.32	1.12–1.56
	DSM-IV	Shanxi	101 schizophrenia trios						0.058	1.20	0.91–1.57
Li^15^	ICD-10	Yuxi	488	53.1	38.5 (10.4)	694	53.5	37.1 (6.8)	0.876	1.01	0.86–1.19
	DSM-IV	Kunming	403	44.4	36.3 (8.7)	604	44.4	36.6 (7.0)	0.489	0.94	0.79–1.12
**Yue**^22^	DSM-IV	Beijing	746	53.1	34.5 (8.7)	1599	52.9	35.8 (7.8)	NA	1.11	0.96–1.29
**Shi**^23^	DSM-IV	Shanghai and Anhui	1238	55.9	36.2 (12.4)	2856	35.5	60.9 (12.2)	0.250	1.06	0.96–1.16
	DSM-IV	Beijing and Shandong	1578	69.8	36.9 (9.3)	1592	50.3	30.8 (11.1)	0.100	0.92	0.83–1.02
	DSM-IV	Guangdong and Guangxi	934	58.4	36.3 (16.6)	2020	47.7	56.1 (13.5)	0.420	0.95	0.85–1.07
Wei^40^	DSM-IV	Guangdong	100	53.0	26.5 (6.9)	69	56.5	25.4 (5.7)	0.064	0.66	0.43–1.02
Chen^14^	ICD-10	Shandong	570	61.5	28.2 (7.8)	448	65.1	23.0 (7.0)	0.013	1.25	1.05–1.49
Liou^24^	DSM-IV	Taiwan	522	55.4	44.1 (9.1)	806	47.5	67.6 (9.4)	0.570	1.05	0.89–1.22
Yang^13^	DSM-IV	Northern China	1024	51.4	27.3 (8.0)	975	49.0	27.7 (8.0)	0.087	1.12	0.98–1.27
Wong^16^	DSM-IV	Sichuan	1086	NA	NA	1060	NA	NA	NA	0.91	0.81–1.02
Wang, 2016	DSM-IV	Jiangsu	1284	63.0	45.8 (11.5)	990	55.4	44.9 (10.1)	0.497	0.96	0.85–1.08
**Bipolar disorder**											
Zhang^21^	DSM-IV	Shanghai	746	NA	NA	762	NA	NA	0.034	1.19	1.03–1.37
Current study	DSM-IV	Shanghai	528	39.7	42.2 ± 10.4	581	40.3	31.9 ± 11.3	0.028	1.21	1.02–1.43

Note: the studies in bold were GWASs. NA, not available; OR, odds ratio; 95% CI, 95% confidence interval.

^1^The N represents the number of individuals having genotyping data for *rs1344706*.
