# Supplementary material for: Genetic association of rs1344706 in ZNF804A with bipolar disorder and schizophrenia susceptibility in Chinese populations
Source: Sci Rep. 2017 Jan 25;7:41140. doi: 10.1038/srep41140 (PMC5264157; doi:10.1038/srep41140)
Supplement: Supplementary Materials [file srep41140-s1.doc]

This document contained Supplementary Materials for:

**Genetic association of *rs1344706* in *ZNF804A* with bipolar disorder and schizophrenia susceptibility in Chinese populations**

Shuquan Rao^1, *^, Yao Yao^2^, Joanne Ryan^3,4^, Chunhui Jin^5^, Yong Xu^6^, Xinhe Huang^1^, Jianxiu Guo^1^, Yueqiang Wen^2^, Canquan Mao^1^, David Meyre^7^, Fuquan Zhang^5,*^

^1^ School of Life Science and Engineering, Southwest Jiaotong University, Chengdu 610031, China

^2^ Department of Fundamental Medicine, Chengdu University of Traditional Chinese Medicine, Chengdu, 611137, China

^3^ Disease Epigenetics Group, Murdoch Children’s Research Institute, & Department of Paediatrics, University of Melbourne, 3052 Parkville, Australia

^4^ Inserm, U1061, Univ Montpellier, Montpellier, France

^5^ Wuxi Mental Health Center, Nanjing Medical University, Wuxi, China

^6^ Department of Psychiatry, First Clinical Medical College/First Hospital of Shanxi Medical University, Taiyuan, 030000, China

^7^ Department of Clinical Epidemiology and Biostatistics, McMaster University, Hamilton, ON L8N 3Z5, Canada

*Correspondence should be addressed to Dr. Fuquan Zhang (Email: zhangfq@njmu.edu.cn) or Dr. Shuquan Rao (Email: [shuquan_rao@163.com](mailto:shuquan_rao@163.com) or raosq@swjtu.edu.cn)

**Contents**

[Supplementary Table 1 …………………………………………………………….…………..3](#__RefHeading___Toc437334868)

Supplementary Table 2………………………………………………….……………………..3

[Supplementary Figure 1……………………………………………………………………….4](#__RefHeading___Toc437334869)

Supplementary Figure 2……………………………………………….………..……………..5

Supplementary Figure 3……………………………………………….………..……………..6

Supplementary Figure 4……………………………………………………………………….8

**Supplementary Table 1 Analysis of allelic association of rs1344706 with BMD characteristics**

| Characeristics | Standard coefficient | *P*-value |
| --- | --- | --- |
| Age of onset | -0.010 | 0.816 |
| Depressive episodes | -0.149 | 0.715 |
| Manic/hypomanic episodes | 0.175 | 0.667 |
| Overall episodes | -/- | -/- |
| Baseline YMRS | 0.170 | <0.001 |

**Supplementary Table 2 Description of the analyzed** samples

| Characteristic | Patients of BD | Controls | *P*-value |
| --- | --- | --- | --- |
| Sample size (*N*) | 537 | 591 | -/- |
| Male (*N*, %) | 213 (39.7) | 238 (40.3) | 0.836 |
| Age (mean ± SD, years) | 42.2 ± 10.4 | 31.9 ± 11.3 | < 0.001 |
| Age of onset (mean ± SD, years) | 33.6 ± 8.3 | -/- | -/- |
| Overall episodes (mean ± SD) | 9.8 ± 10.2 | -/- | -/- |
| Depressive episodes (mean ± SD) | 5.5 ± 6.9 | -/- | -/- |
| Manic/hypomanic episodes (mean ± SD) | 4.3 ± 5.1 | -/- | -/- |

**Figure S1 Funnel plot with pseudo 95% confidence limits for meta-analysis of *rs1344706***


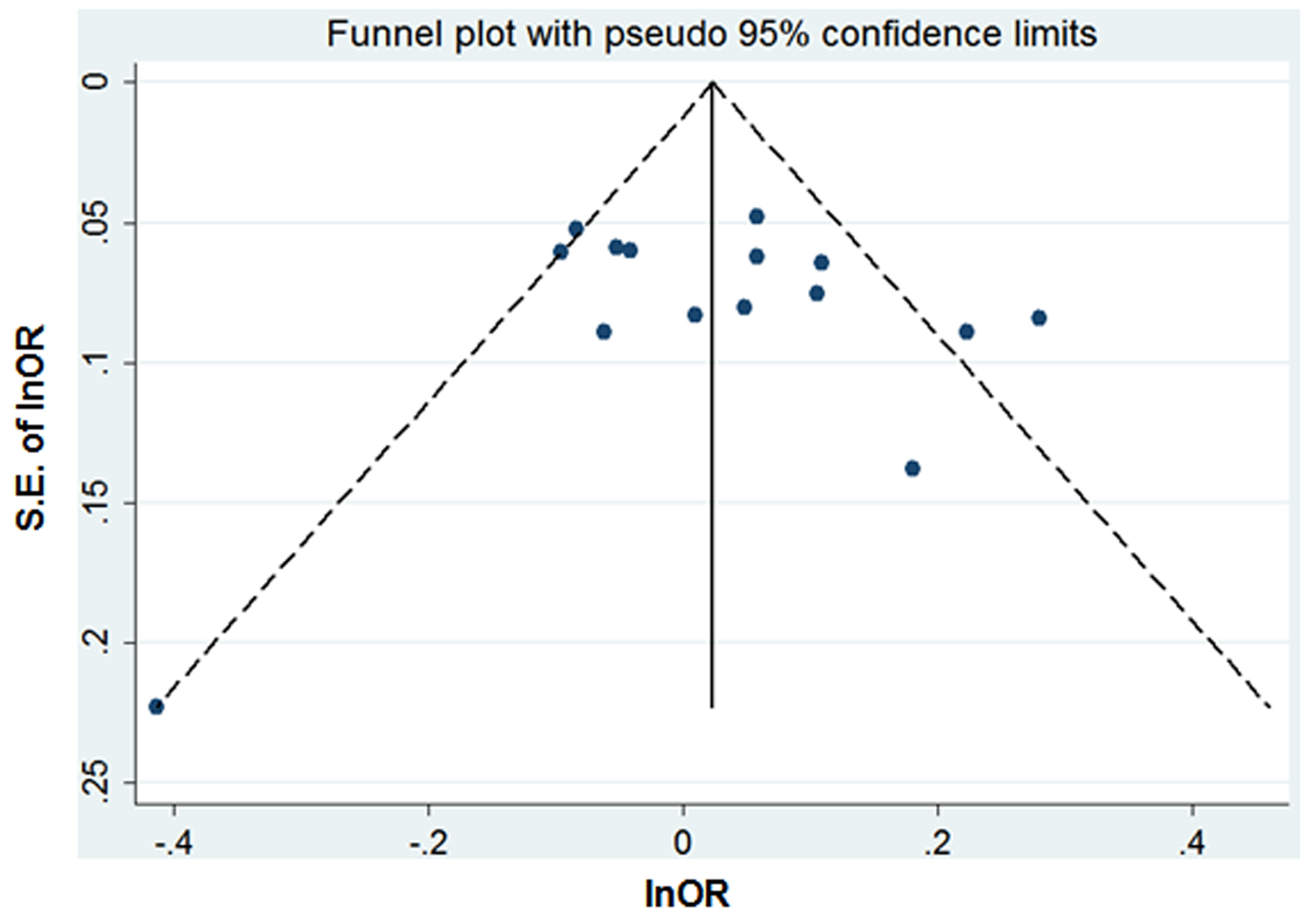


**Fig. S2 Meta-analysis for *rs1344706* T-allele with schizophrenia in Northern, Central and Southern Chinese populations**


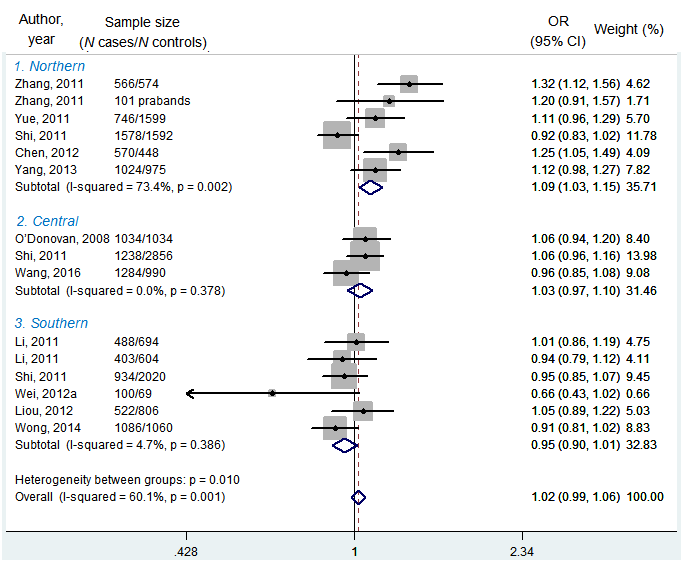


The random-effect model was applied to pool the data for Northern Chinese populations, while the fixed-effect model for Central and Southern Chinese populations.

**Fig. S3 Allele frequency distributions of the risk SNP *rs1344706* in global populations**


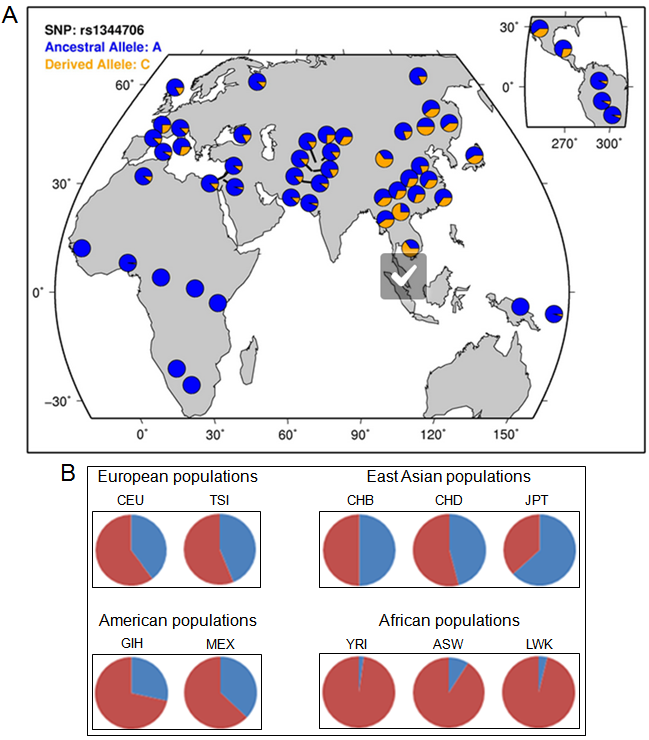


(A) The derived allele (C-allele) of *rs1344706* showed dramatic frequency differences among 53 world populations. The map was generated from HGDP selection browser, which was one online software ([*http://hgdp.uchicago.edu/cgi-bin/gbrowse/HGDP/*](http://hgdp.uchicago.edu/cgi-bin/gbrowse/HGDP/))^1^.

(B) Allele frequency distributions of *rs1344706* from the 1000 genomes projects. CEU, Utah residents with Northern and Western European ancestry; TSI, Toscans in Italy; CHB, Han Chinese in Beijing; CHD, Chinese in Metropolitan Denver; JPT, Japanese in Tokyo; GIH, Gujarati Indians in Houston; MEX, Mexican ancestry in Los Angeles; YRI, Yoruba in Ibadan; ASW, African ancestry in Southwest USA; LWK, Luhya in Webuye, Kenya.

**Figure S4 Expression profiling of *ZNF804A* in 61 mouse tissues**

**
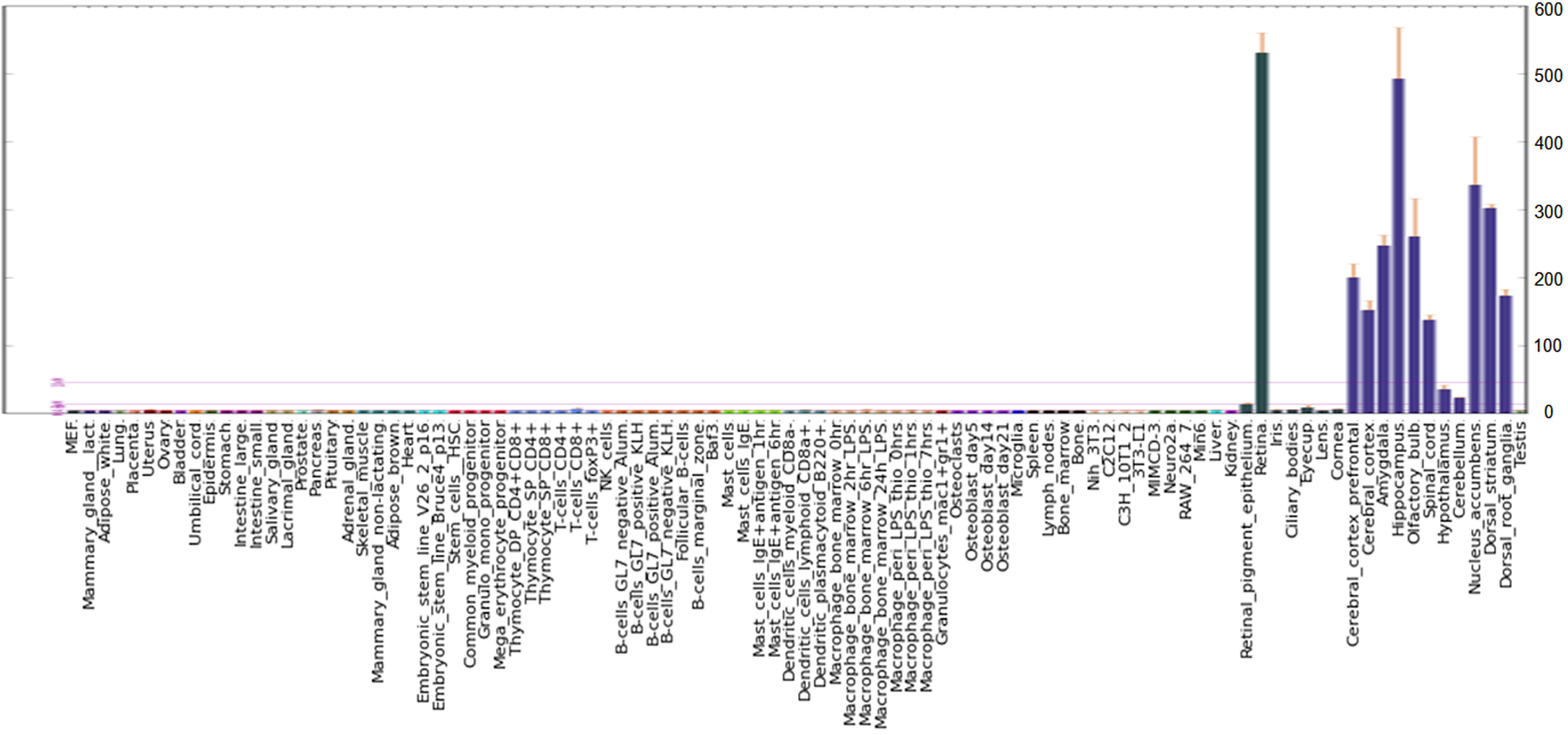
**

Note: *ZNF804A* is mainly expressed in the central nervous system, with the highest expression level in hippocampus (data from the BioGPS, [*http://biogps.org/*](http://biogps.org/))^2^. X-axis represented different tissues, and Y-axis indicated the normalized expression level of ZNF804A across all tissues.

**Supplementary references**

**1.** Pickrell, J.K. et al. Signals of recent positive selection in a worldwide sample of human populations. *Genome Res* 19, 826-837 (2009).

**2.** Wu, C., Jin, X., Tsueng, G. Afrasiabi C, Su AI. BioGPS: building your own mash-up of gene annotations and expression profiles. *Nucleic Acids Res* 4(D1), D313-316 (2016).
